# Transferring skills in quality collaboratives focused on improving patient logistics

**DOI:** 10.1186/s12913-018-3051-8

**Published:** 2018-04-02

**Authors:** Anne Marie Weggelaar-Jansen, Jeroen van Wijngaarden

**Affiliations:** Erasmus School of Health Policy & Management, Erasmus University Rotterdam, Campus Woudestein, P.O. Box 1738, 3000 DR Rotterdam, The Netherlands

**Keywords:** Quality improvement, Quality improvement collaborative, Patient logistics, Advanced access, Process redesign, Education

## Abstract

**Background:**

A quality improvement collaborative, often used by the Institute for Healthcare Improvement, is used to educate healthcare professionals and improve healthcare at the same time. However, no prior research has been done on the knowledge and skills healthcare professionals need to achieve improvements or the extent to which quality improvement collaboratives help enhance both knowledge and skills. Our research focused on quality improvement collaboratives aiming to improve patient logistics and tried to identify which knowledge and skills are required and to what extent these were enhanced during the QIC.

**Methods:**

We defined skills important for logistic improvements in a three-phase Delphi study. Based on the Delphi results we made a questionnaire. We surveyed participants in a national quality improvement collaborative to assess the skills rated as 1) important, 2) available and 3) improved during the collaborative. At two sense-making meetings, experts reflected on our findings and hypothesized on how to improve (logistics) collaboratives.

**Results:**

The Delphi study found 18 skills relevant for reducing patient access time and 21 for reducing throughput time. All skills retrieved from the Delphi study were scored as ‘important’ in the survey. Teams especially lacked soft skills connected to project and change management. Analytical skills increased the most, while more reflexive skills needed for the primary goal of the collaborative (reduce access and throughput times) increased modestly.

At two sense-making meetings, attendees suggested four improvements for a quality improvement collaborative: 1) shift the focus to project- and change management skills; 2) focus more on knowledge transfer to colleagues; 3) teach participants to adapt the taught principles to their own situations; and 4) foster intra-project reflexive learning to translate gained insights to other projects (inter-project learning).

**Conclusions:**

Our findings seem to suggest that Quality collaboratives could benefit if more attention is paid to the transfer of ‘soft skills’ (e.g. change, project management and communication skills) and reflexive skills (e.g. adjusting logistics principles to specific situations and inter-project translation of experiences).

**Electronic supplementary material:**

The online version of this article (10.1186/s12913-018-3051-8) contains supplementary material, which is available to authorized users.

## Background

Teaching professionals how to improve daily practices to achieve cost reductions and enhance quality and safety has been on the healthcare agenda [1]. A common improvement method is the quality improvement collaborative (QIC), often used by the Institute of Healthcare Improvement (IHI) [2, 3]. Most QICs strive for a combination of substantial improvements in quality of care, optimized patient logistics, safe working routines and patient-centeredness [4–10] by implementing best practices and the latest scientific insights (e.g. clinical guidelines) [7, 8, 11]. Øvretveit et al. ([2] p. 345) define a QIC as “a collaborative [that] brings together groups of practitioners from different healthcare organizations to work in a structured way to improve a specific aspect of the quality of their service. It involves them in a series of meetings to learn about best practices in the chosen area, about quality methods and change topics and to share their experiences of making changes in their own local setting.” Øvretveit et al. [2] emphasize the importance of learning/teaching skills for healthcare professionals in the work on a specific project. Most QICs use the IHI Breakthrough methodology to educate healthcare professionals [2, 3, 12]. However, little is known about the skills professionals need to successfully improve their daily practices and if indeed these skills are taught and developed in QICs [13]. Our study aims to address this gap in knowledge.

Our study focused on QICs aimingr to optimize patient logistics, which encompasses “the complicated set of decisions related to the physical movement of patients throughout the healthcare chain (acute settings and post-acute care)” ([14] p. 155). Optimizing patient logistics involves “the analysis, design, planning, and control of all of the steps necessary to provide a service for a client” ([15] p. 1). Research shows that poor patient flow management results in long access times, queues, delays, long stays for patients, workload variability for healthcare professionals, supply shortages, wasted resources and low levels of productivity [16–18]. Several logistic improvement studies report increased quality of care, services and operational efficiencies obtained by reducing waste and costs, and preventing medical errors [19–21]. QICs aim to spread such best practices.

We studied two QICs: Advanced Access (AA), aimed at reducing access time to the outpatient clinic to a maximum of two days [22] and Process Redesign (PR), aimed at reducing throughput time for admitted patients by at least 20% [23]. Both QICs used the principles and examples that had been successfully tested in other settings and countries. Both QICs tried to teach participants how to identify and resolve bottlenecks in patient flow, using these principles. AA taught participants how to balance demand and supply in the out-patient clinic to reduce waiting times. Most participants think that waiting times are caused by shortages in supply, whereas the main cause is often backlog, because supply is not responsive enough to changes in demand. Participants were taught how to make their supply more flexible and adaptive to demand, by reducing the number of ‘fixed slots’ in their schedules, for example. PR taught the participants to identify delay and bottlenecks in healthcare processes. Participants learned how to make a radical new process for delivering healthcare, aiming to achieve major improvements. In addition, the participants were taught to assign the right healthcare professionals for the task to seamlessly execute patient processes.

The educational approach of the studied QICs organized learning sessions taught by change experts and experts on subjects related to the improvement aim, supported by change packages (e.g. booklets describing the ten distinctive phases combined with PowerPoint files and communication tools) [24]. They also arranged connections to a collaborative extranet for sharing information and QIC staff visited participants on site to provide support [24].

The objective of this study is to identify if QICs provide professionals with the necessary skills to successfully improve healthcare practice and how this could be optimized. Our research question was: “How can a QIC optimize the development of healthcare professionals’ skills to improve patient logistics?” This was divided into three sub-questions: 1) What skills do teams need to make logistic improvements? 2) To what extent are these skills available and improved during the QIC? 3) How can the educational program of a QIC be improved to enhance skills development?

## Methods

**Fig. 1:**
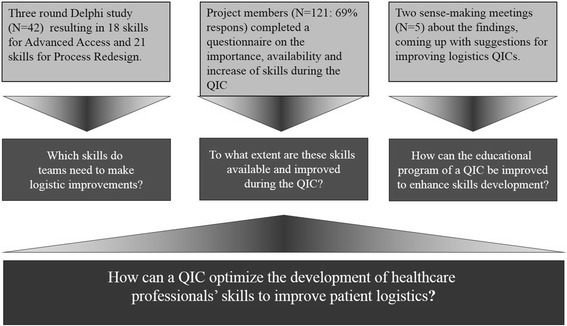
Overview of research methodology

We used mixed methods, organized in three stages (see overview in Fig. 1), to answer our research question. First, a Delphi study to identify the skills participants need to improve patient logistics in their practice. Second, an assessment of team members’ skills, using a questionnaire based on the Delphi study results [25]. Third, we held two sense-making meetings [26] with experts to reflect on the findings and identify ways to improve the QICs educational program. The two QICs run for five years in the Netherlands. Twenty-four out of the approximately 110 Dutch hospitals participated, divided into three tranches of eight hospitals. Each hospital was represented by two or three units (i.e. wards, out-patient services or teams). We studied the last two tranches of 16 hospitals, during the last three years of the project.

Ethics approval for this study was not necessary under Dutch law as no patient data was collected; consent was obtained from the involved participants and respondents.

### Delphi study

Since there is literally no evidence available on skills needed for logistical improvement projects in healthcare, we started the Delphi using expert opinions. Six QIC staff members: three logistic scientific experts and three change experts assigned to the AA or PR QIC being responsible for learning sessions and site visits independently provided statements. Subsequently, two researchers independently arranged these statements into clusters, resulting in a synthesized list of skills. This task was done by 1) deduplicating the same statements, 2) clustering closely related statements, 3) and having the two researchers discuss the clustering to reach consensus. The outcome of this process was discussed with the QIC leaders for member-checking reasons and resulted in a further reduction of statements by providing the researchers better descriptions of skills.

Next, we used three iterative rounds of the Delphi process to convert these two lists into group consensus [25, 27, 28]. Anticipating that a heterogeneous panel would lead to better results than a single-specialty panel [29–33], we selected 47 potential panel members from various backgrounds [34], including 17 researchers working on logistic improvement projects from (inter)national publications, national networks and other experts’ suggestions and 30 Dutch practice experts with experience in logistics improvement projects who were regarded as experts in their hospital.

Since anonymity is crucial in the Delphi method [29–31, 35] we used the BCC field in e-mails to prevent participants discovering the identity of others. In all three rounds, respondents were asked: Which of the following skills do you consider important for a logistics improvement project? The respondents graded each skill on a ten-point Likert scale (ranging from 1 not important to 10 most important), adding comments to substantiate their grade, suggest new skills or reformulations of the listed skills.

We computed the medians and conducted a Fleiss kappa test [36, 37] with a 95% confidence interval to test inter-rater reliability. A skill stayed the same if the resulted in a median of 8 or more; between 5 and 8 we reformulated a skill, and below 5 we removed it. Between the rounds the two researchers discussed upon consensus the respondents’ comments and their suggestions for reformulation. After three rounds, all skills were above 8.0.

### Questionnaire

We developed two questionnaires based on the results of the Delphi study, one for the AA QIC and one for the PR QIC. Both questionnaires included skills for logistics improvement projects in general and skills specific to the aim of the QIC project (see for questionnaires the Additional file 1). All 176 project team members were included in the study (96 in AA and 80 in PR). Project teams came from eight hospitals; one academic (six teams) and seven general (20 teams) of which five were teaching hospitals (16 teams). The questionnaire was distributed at the final QIC meeting and sent to those not present. The respondents were asked to score on a five-point Likert scale (ranging from 1 not important/available/increased, to 5 very important/available/increased):

- how important each skill was for their ability to improve their logistics processes
- whether the skill was available in their team
- whether they increased the skill by participating in the QIC or improvement project.

All questionnaires were returned anonymously and the data was analyzed with SPSS 19 using descriptive statistics: frequency counts, sum scores and percentages. All items were screened for univariate and bivariate normality to detect outliers; no extreme values were found. Data was missing in 3% of the items. ANOVAs (*p* < 0.5) were performed to examine if differences in professional background and role in the project led to different outcomes in the participants’ ratings for skills availability and improvement during the QIC. The internal consistency of each questionnaire was assessed by computing Cronbach’s *α* (range 0.87–0.97). Pearson product-moment correlation coefficients of the sum scores were calculated to assess if there was a relationship between importance, availability in the team and increase in skills.

### Sense-making meetings

To interpret the results, we held two sense-making meetings [38–40] with two professors in health logistics, two members of the logistic QIC staff and the overall QIC program leader (referred to as attendees, *N* = 5). The aim was to understand why QICs did or did not contribute to an increase in skills and to identify how QICs can be improved. Applying sense-making methods can address ‘how’ and ‘why’ questions and explore the complex relationships that underlie our findings [41].

We asked the attendees to share their opinions, thoughts and experiences about our findings. During discussions, one researcher challenged each attendee to explicitly examine underlying perceptions and beliefs about the skills needed, transfer of skills in the QIC and the implications for logistic improvement work within hospitals. The ladder of inference, as a tool, was used to foster open scrutiny of the underlying perceptions and beliefs of the participants [42]. The ladder first maps how we move from observable data to selecting data. The next rungs attach meaning to data and make assumptions based on these meanings, followed by drawing conclusions that steer action, which in turn affects data [43, 44]. The aim of using the ladder of inference [45] was to help attendees to:

- become aware of their thinking and reasoning (reflection);
- make their thinking and reasoning apparent to others (advocacy);
- gain understanding of other’s thinking and reasoning (inquiry);
- prevent jumping to conclusions.

The sense-making meetings resulted in narratives that illustrated the attendees’ experiences, perceptions and beliefs (bottom step of the ladder of inference) about the problems the QIC faculty faces. The attendees shared their understanding of what could be improved and why. After the second meeting saturation occurred.

We took notes on flip charts during the meetings, which were audiotaped and transcribed. Transcriptions and flip charts were analyzed deductively (related to the research questions) and inductively (based on themes emerging from the data). Our findings were sent to five attendees for member-checking.

## Results

### Stage 1: Delphi study

The experts provided 272 statements on skills relevant to improving patient logistics (100% response, *M* = 47 statements, range 26–87 statements). After clustering two lists of skills remained: for AA (28 skills) and PR (26 skills). Both lists shared 14 general skills for logistics improvements. The remaining skills were specific to the aim of the particular logistic improvement. Most, however, were variations of the same topic.

Of all invited respondents, 17 experts (100%) and 25 practice experts (83%) agreed to participate (see Table 1). This number (42) exceeds the recommended panel size of at least 30 respondents [30]. The response rate in rounds one and two was 100% (*N* = 42); two practice experts withdrew in round three (*N* = 40, response 96%).

**Table 1:** Characteristics of Delphi panel experts (*N* = 42)

Characteristics	Category	Expert group
Gender	Male	12
	Female	30
Age	< 30 year	8
	30–40 years	14
	41–50 years	11
	51–65 years	9
Professional background	Advisers/policy makers	8 (2 are also researchers)
	Medics	8
	Nurses	6
	Management	3
	Outpatient clinical staff	7
	Applied healthcare staff	4
	Researchers	8 (2 are also advisers)
Years of experience in logistics improvement	< 2 years	6
	2–5 years	11
	6–10 years	15
	> 10 years	10
Specialty (source of expertise)	Consultancy	6
	Project leader	8
	Research	8
	Research and consultancy	2
	Practice in projects	18

Based on computed median results after the first Delphi round, nine skills were reformulated, eight were clustered, seven were omitted (classified as unimportant by scores below 5) and two new skills were added based on five suggestions. In the second round, three skills were reformulated and one of the new skills was clustered; the other new skill was scored above the 8 threshold. In the third round, only four minor reformulation changes were made and no skills were added or omitted. The Fleiss Kappa resulted in a substantial agreement with 0.83. The Delphi study resulted in a list of 18 skills for AA and 22 skills for PR. See Table 2 for the skills lists; gray shading shows the similarities.

**Table 2:**
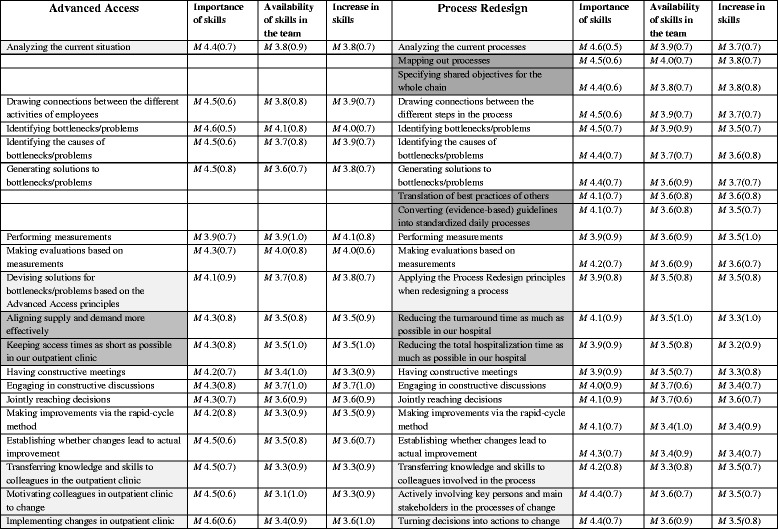
Skills for advanced access and process redesign

Assessment of Importance, Availability in the team, and Increase in skills

Legend: *M* = Mean; (Standard Deviation); The white cells show the general skills, the light gray cells are slight adjustments based on the aim of the logistic improvement project and the dark gray cells are completely different skills

### Stage 2: Survey

Next, using the results of the Delphi study, we surveyed each QIC to examine the importance, availability and increase in skills.

#### Respondents

Fifteen AA teams and 11 PR teams participated and completed 121 of the 176 questionnaires (69% response): 70 for AA (73% response) and 51 for PR (64% response). Respondents included medics (e.g. physicians, fellows and residents), nurses (e.g. registered nurses, nursing students and nurse practitioners), allied healthcare professionals (e.g. ambulatory physicians, respiratory, physical and occupational therapists, dieticians and pharmacists), administrative employees supporting care planning, management and other staff (e.g. advisers and policy makers) (see Table 3). The respondents were representative for gender, age and roles in the project team.

**Table 3:** Characteristics of survey respondents

	Advanced access		Process redesign	
	*N* = 70	Percentage	*N* = 51	Percentage
Gender				
male	16	22.9%	22	43.1%
female	54	77.1%	29	56.9%
Age				
< 30 years	10	14.3%	5	9.8%
31 to 40 years	15	21.4%	18	35.3%
41 to 50 years	35	50.0%	18	35.3%
> 50 years	10	14.3%	10	19.6%
Project team role				
support staff	12	17.1%	10	19.6%
project team member	40	57.1%	25	49.0%
project leader	14	20.0%	11	21.6%
other	4	5.7%	3	5.9%
absent			2	3.9%
Professional background				
management	22	31.4%	12	23.5%
administrative employees	14	20.0%	1	2.0%
supporting staff	12	17.1%	13	25.5%
allied health care staff	8	11.4%	0	0.0%
nursing	5	7.2%	8	15.7%
medic	5	7.2%	15	29.4%
other	4	5.7%	2	3.9%

#### Importance of skills

The respondents regarded most skills as important, resulting in mean scores above four on the five-point Likert scale (*M* between 4.0 and 4.6 for AA and 3.9 and 4.7 for PR). Analytical skills were seen as most important while skills related to measurements were least important. Surprisingly, specific skills connected to the aim of the QIC were generally assessed as moderately important; namely, for PR: “reducing both total hospitalization time and turnaround time” and for AA: “skill of devising solutions for bottlenecks/problems based on the AA principles” (see Table 2, column Importance).

No significant statistical differences were seen depending on the professional background (*F*(3,46) = 1.36, *p* = 0.27) and between project roles (*F*(2,45) = 2.57, *p* = 0.09).

#### Availability of skills in the project team

For both QICs, respondents felt that the skills related to change management were most lacking. In contrast, analytical skills were seen as more available in the team.

No statistically significant differences were found in availability of skills for project role (*F*(2,44) = 0.18, *p* = 0.83) and profession (*F*(3,45) = 1.26, *p* = 0.30). Nevertheless, the skill of making improvements via the rapid-cycle method in PR showed a remarkable but not statistically significant difference; project leaders rated this skill (*M* = 3.9, *SD* = 0.7) and project team members as (*M* = 3.2, *SD* = 0.5).

#### Increase in skills

Overall, the assessment of the degree of increase in skills was lower in PR (*M* between 3.2 and 3.8) than in AA (*M* between 3.6 and 4.3). See Table 2, column Increase. The skills related to engaging other colleagues showed the least increase for both QICs; “motivating colleagues” for AA and “actively involving key persons and main stakeholders in the process of change” for PR and “transferring knowledge and skills to colleagues” for both QICs. The two QICs assessed the increase in analytical skills (e.g. the ability to conduct measurements) differently. AA acquired the directly necessary change management skills only slightly: e.g. “motivating colleagues” and “transfer of knowledge and skills to other colleagues”. The skills directly connected to the aim of the PR QIC were acquired even less: “reducing hospitalization time” and “reducing turnaround time as much as feasibly possible in our hospital”.

No significant effect in the assessment of increase of skills was found for project role (*F*(2,39) = 0.35, *p* = 0.71) and professional background (*F*(3,40) = 0.68, *p =* 0.58).

#### Combination of availability and increase in skills

In both QICs, all correlation coefficients were positive and moderate in terms of strength (ranging from 0.29 to 0.35). Both QICs showed an exceptionally strong association between availability and increase in skills (see Table 4). Thus, if availability of skills in the team was perceived as low, the increase in this skill during the QIC was also rated as low and vice versa for high perceived availability and increase. This suggests that skills already present are developed further during the QIC and skills that are barely available were less developed.

**Table 4:** Correlation between importance, availability and increase in skills

	Availability	Increase
Advanced access (*N* = 70)		
• Increase	*r* = 0.35	
	*p* < 0.01	
• Availability		*r* = 0.801
		*p* < 0.0001
Process Redesign (*N* = 51)		
• Increase	*r* = 0.29	
	*p* = 0.04	
• Availability		*r* = 0.61
		*p* < 0.001

### Stage 3: Sense-making meetings

We present the results of the two sense-making meetings on the Delphi/survey findings in terms of the attendees’ four key problems and suggestions to improve QICs.

#### Problem 1: No project and change management skills

**Fig. 2:**
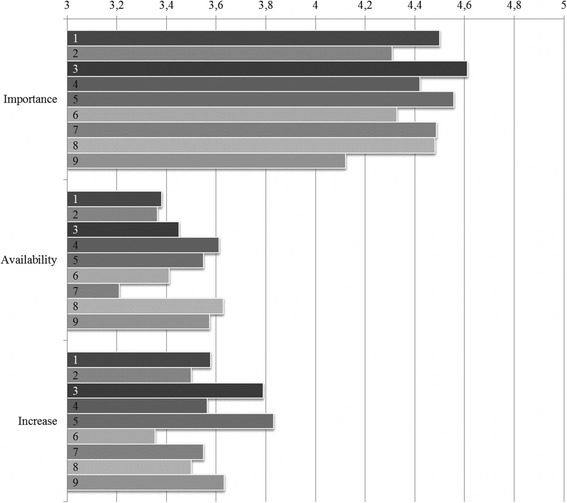
Change management skills considered Important, skills Available in project team and Increase in skills during QIC. 1 – AA: Transferring knowledge and skills to other colleagues at the outpatient clinic. 2 – PR: Transferring knowledge and skills to other colleagues involved in the process. 3 – AA: Implementing changes at the outpatient clinic. 4 – PR: Turning decisions into action for change. 5- AA: Establishing whether changes lead to actual improvement. 6 - PR: Establishing whether changes lead to actual improvement. 7 - AA: Motivating colleagues at the outpatient clinic. 8 - PR: Actively involving key persons and stakeholders in the change processes. 9 - PR: Translation of best practices of others

The Delphi results showed that not only skills for identifying and finding solutions for logistics problems are important, but also project and change management skills. Despite their importance, both availability (*M* ranging from 3.2 to 3.8) and increase in these skills were rated low (*M* ranging from 3.4 to 3.8). See Fig. 2 for an overview of change management scores.

The attendees considered change and project management skills hard to teach. They see them as personal competences developed over time. Knowledge in this area is not just ‘solid know-how’, but also connected to context and understanding: ‘know-why’ and ‘knowing-what-to-do’. Know-how’ was shared via lectures and change packages that explained concrete improvement steps. ‘Know-why’ and ‘know-what-to-do’ are based on understanding interactions between people. This is harder to teach. Attendees also questioned whether healthcare professionals need to be highly skilled in project and change management. All should understand the basics, but it would be more efficient to select the right mix of project team members, ensuring that these skills are available to the team.

#### Problem 2: Knowledge should be transferred to the whole system

It is essential to spread the gained knowledge to other healthcare employees because, according to the attendees, *all* hospital employees involved in the improvements should develop skills, not just project team members. Engaging other employees is seen as vital at several stages, especially in rapid-cycle experiments (part of Breakthrough change methodology). These experiments have blurred the boundaries between project work and daily routines. Hence, it is important that team members share their aim, experiment, methodology, measures and so forth with all employees. However, the survey showed that respondents felt only modestly capable of involving key persons/stakeholders in transferring knowledge and skills to other colleagues. Therefore the attendees suggested that QICs should pay more attention to communication skills so that participants can deal with the various perspectives and languages of the stakeholders.

#### Problem 3: The principles listed in the change package did not fit the problems faced

QIC participants felt that their skills increased very modestly. Attendees of the sense-making meetings concluded that what is taught during the QIC does not always fit the needs and expectations of participants. Information was transferred in ‘bite-sized chunks’, e.g. the ten distinctive phases. This knowledge helped the project teams get started, but they soon needed to adapt it to make it fit their context. Most teams lacked the skills to do this and, as the survey showed, these skills increased only modestly (see Table 5).

**Table 5:** Availability/Increase in skills needed to adjust daily practice to reach goals

Advanced access			Redesign process		
	Available	Increase		Available	Increase
Aligning supply and demand more effectively	*M* 3.5 (0,8)	*M* 3.5 (0.9)	Reducing turnaround time as much as possible in our hospital	*M* 3.5 (1.0)	*M* 3.3 (1.0)
Keeping access times as short as possible for our outpatient clinic	*M* 3.5 (1.0)	*M* 3.5 (1.0)	Reducing total hospitalization time as much as possible in our hospital	*M* 3.5 (0.8)	*M* 3.2 (0.9)

Legend: *M* = Mean; (Standard Deviation)

The attendees suggested using a team-centered educational approach, focusing on what members want to learn, rather than on what faculty thinks they should teach. The assumption is that participants, as learners, differ in their motivation, needs, interests and the skills they wish/need to develop. Their context differs and this requires teaching that is connected to the problems they face. This educational approach challenges QIC faculty to understand what a team wants to do in their own organization and what they need for this.

#### Problem 4: Overemphasis on project goals instead of continuous improvement

The attendees argued that the QICs focused too much on reaching project targets and too little on developing skills that QIC participants need. After the QIC concludes, teams should be able to deal with an array of logistical challenges, also in future situations. Surprisingly, the increase in the skills connected to the primary aim of the QICs scored very low (see Table 5), suggesting that this was not the case at the end of the QIC.

The attendees concluded that participants learned only the basics. Faculty should pay more attention to inter-project learning. Providing tools for inter-project learning teaches participants to translate knowledge and experiences from one project to another or to similar problems or situations. Sharing what they learn explicitly with others, could include writing reviews and discuss them with other teams, organizing reflective meetings to share the lessons learned, and organizing brainstorming meetings to discuss (potential) problems and their solutions.

## Discussion

In the first stage of this research we identified the skills teams need to work on quality improvement by focusing on two patient logistics QICs; Advanced Access (AA) and Process Redesign (PR). A Delphi study produced lists of 18 skills for AA and 22 skills for PR. Our findings show many similarities with Gammelgaard and Larson’s study [46] that distinguished four categories of skills for logistics managers: people skills, analytical skills, technical logistics skills and (change) management skills. Also, in the Health Foundation paper on improvers’ habits the importance of a mix of conceptual (‘habits of mind’) and practical skills (‘habits of persons) and for the latter the communication skills to co-produce are stressed [47].

In the second phase we studied the availability of these skills and increased during the QICs. The survey respondents rated most skills modestly available. The skills related to people and change management, especially ‘transferring knowledge and skills to other colleagues involved in the process’, were seen as less available in project teams than ‘harder’ technical logistic and analytical skills, such as measuring and process analysis. Overall, skills increase was rated as very modest. Prajogo and Sohal [48] conclude that technical logistic knowledge and analytical skills are essential, but this knowledge has no significant impact on daily practice improvement work. They found that skills connected to people and change management are more important. Other studies show that teaching change methods to healthcare professionals is a key success factor in achieving change [49–51]. In addition, project management skills (e.g. meeting facilitation) are regarded as most important by Le May et al. [45] and Thai [52], who both studied the training needs of supply chain managers.

This brings us to the final question of this study: How can we improve a QIC educational program to enhance the transfer of necessary skills? No study to date has demonstrated that involvement in a QIC enhances skills in logistics improvement techniques, or in change and project management [13, 53, 54]. The attendees of the sense-making meetings identified four key problems related to skills development in QICs and how they influence the sustainability of improvement work in healthcare. The attendees suggested that more emphasis should be given to skills required to transfer knowledge to colleagues, to adapt the taught principles to their own situation, and to inter-project translation of knowledge. Numerous studies show that the key to continuous improvement work in daily practice is developing the skills of all professionals involved [7, 55, 56]. Pronovost states: “Many quality improvement projects often fail to achieve their goals… An even larger number of projects fail because of adaptive challenges.” ([57] p. 560). The attendees suggested that tapping into the needs of participants and fostering intra-project learning would harvest more implicit knowledge that could thus be shared with others. They suggested that QICs could give more attention to what goes well and what should be done differently in future improvement projects.

We expect these suggestions and aspects of our other findings to be relevant to other (non-logistic) QICs as well. Many of our findings and all the suggestions do not concern technical logistic skills that much, but focus on skills relevant to introducing quality improvements in general, such as project and change management and communication skills. Not all of these need be taught in a QIC, but they need to be available in the participating teams. Given that professionals need to change their daily practice on demand to reduce costs and improve quality of care, we think that these non-technical skills should become an integral part of their primary training and continuing medical education [58].

### Limitations of the research

#### Delphi

Since its inception, there is no consensus on how to conduct a Delphi study [26, 59], or on the value of group consensus [60]. We boost validity and credibility by following the protocol of Boulkedid et al. [31] and Okoli et al. [61]. Still, respondent quality determines the Delphi outcome. Our wide range of respondents with varying experience and relevant expertise, enlarges the likelihood that our results are relevant across multiple contexts and settings. Nevertheless, important skills could still be lacking. Furthermore, the skills clustering was done primarily by the two authors and only checked by the experts. Although the experts agreed with the final clustering, concept mapping would have been a more rigorous approach to identifying the relevant clusters.

#### Survey

The questionnaire did not undergo formal psychometric testing; only Cronbach’s ɑ was calculated. Each item scored highly in all three questionnaires (between 3.6 and 4.9) so the distinctions of a five-point Likert scale could be too broad. We recommend using a ten-point Likert scale. Our data is based on self-assessment with a possible bias toward socially desirable answers. And, due to the nature of improvement work, the outcome (i.e., enhanced skills) could be affected by other activities.

#### Sense-making meetings

The two sense-making meetings, attended mostly by QIC faculty, were perhaps biased. However, the attendees are considered experts in both logistics and/or QICs and shared their knowledge and experience to identify the practical implications of our findings. Several researchers describe the benefits and rigor of sense-making as a research method [38] resulting in an interaction between researchers and practitioner [49]. However, it is not commonly used in healthcare research and may be prone to subjectivity.

## Conclusions

Our study shows that QIC participants experience only a modest increase in skills. Teams particularly lack project and change management skills. Analytical skills increase the most, while skills needed for the primary collaborative goal increase modestly. QIC methodology assumes that healthcare organizations can be changed on the whole by teams that adopt new ideas and become competent in using improvement techniques in their own context [15]. With their educational components, QICs are supposed to support the professional’s learning process and transform healthcare organizations [58]. Our results lead us to question if this is the case.

The sense-making meeting attendees suggested improving the educational components of the QIC. The focus should shift to project and change management skills, rather than the principles behind the best practices. These skills relate to the transfer of skills to other colleagues, to adapt the taught principles to own situations, and intra-project learning to translate gained insights to other projects. To support this the attendees called for a shift toward process-oriented transfer.

## Additional files

Additional file 1: Questionnaire used to assess the skills seen as 1) important, 2) available and 3) improved during the QICs. (DOC 103 kb)

## Abbreviations

AA: Advanced Access
IHI: Institute for Healthcare Improvement
PR: Process Redesign
QIC: Quality Improvement Collaborative
