# Supplementary material for: Transferring skills in quality collaboratives focused on improving patient logistics
Source: BMC Health Serv Res. 2018 Apr 2;18:224. doi: 10.1186/s12913-018-3051-8 (PMC5879809; doi:10.1186/s12913-018-3051-8)
Supplement: Supplementary file 1 — Questionnaire used to assess the skills seen as 1) important, 2) available and 3) improved during the QICs. (DOC 103 kb) [file 12913_2018_3051_MOESM1_ESM.doc]

# Additional file 1: Questionnaire to assess learning skills – related to paper BHSR-D-18-00009R1

## Introduction

To achieve good results for your project it is important to acquire the relevant skills. To gain insight into how much you learn we have developed this questionnaire (based on a Delphi study). We are using this questionnaire to understand more about the learning style that suits you and your team (‘how do we learn?’)[[1]](#footnote-2) and the skills important to Process Redesign (‘what are we supposed to learn?’).

Please complete the questionnaire and leave it in the envelope on the table. We have provided a copy (and envelope) for any team members not present today. If they return their copy to us before X, we will include them in the analysis of your team. Your project leader will receive an overview of the scores and our recommendations on which skills are important for you to develop.

We are also using the data from this questionnaire to study how people learn on quality collaboratives. It goes without saying that the data will be processed anonymously. If you object our using your data for this research, please inform project leader X. You may also contact her if you have any questions.

## Personal Details

**Hospital:**

**Team:**

**Gender:**  Male

Female

**Year of birth:**

**What is your role in the team?**   Project leader

Project team member

Adviser / project secretary

Other … (please explain)

**What is your job in the hospital?**  Physician, fellow or resident

RN, nursing student or nurse practitioner

Ambulatory physician or physical therapist or

occupational therapist or dietician or pharmacist

Administrative or supporting care planning staff

Manager

Adviser or policy maker

Other….

**What is your highest level of education?**  LBO

MBO

HBO

University

Other….

**How many years of working experience?**  0–5 years

6–10 years

11–15 years

more than 15 years

## Skills in PR collaborative

These questions are about the skills that a team must have in order to implement a PR project successfully. For each listed skill, please indicate your opinion of its importance, ranging from 1 (not), 2 (slightly), 3 (moderately), 4 (important), to 5 (very important).

| 16 | **How important are the following skills for a successful PR project?**  **The skills to…** |  | 1 t/m 5 |
| --- | --- | --- | --- |
|  | Analyzing the current processes |  |  |
|  | Mapping out processes |  |  |
|  | Drawing connections between the different steps in the process |  |  |
|  | Identifying bottlenecks/problems |  |  |
|  | Identifying the causes of bottlenecks/problems |  |  |
|  | Specifying shared objectives for the whole chain |  |  |
|  | Generating solutions to bottlenecks/problems |  |  |
|  | Performing measurements |  |  |
|  | Making evaluations based on measurements |  |  |
|  | Making improvements via the rapid-cycle method |  |  |
|  | Establishing whether changes lead to actual improvement |  |  |
|  | Having constructive meetings |  |  |
|  | Engaging in constructive discussions |  |  |
|  | Jointly reaching decisions |  |  |
|  | Turning decisions into actions to change |  |  |
|  | Applying the Process Redesign principles when redesigning a process |  |  |
|  | Reducing the turnaround time as much as possible in our hospital |  |  |
|  | Reducing the total hospitalization time as much as possible in our hospital |  |  |
|  | Translation of best practices of others |  |  |
|  | Converting (evidence-based) guidelines into standardized daily processes |  |  |
|  | Transferring knowledge and skills to colleagues involved in the process |  |  |
|  | Actively involving key persons and main stakeholders in the processes of change |  |  |
|  | Any other skills that are important but not mentioned? |  |  |

| 17 | **How do you asses the availability of skills in your team?**  **The skill to…** |  | 1 t/m 5 |
| --- | --- | --- | --- |
|  | Analyzing the current processes |  |  |
|  | Mapping out processes |  |  |
|  | Drawing connections between the different steps in the process |  |  |
|  | Identifying bottlenecks/problems |  |  |
|  | Identifying the causes of bottlenecks/problems |  |  |
|  | Specifying shared objectives for the whole chain |  |  |
|  | Generating solutions to bottlenecks/problems |  |  |
|  | Performing measurements |  |  |
|  | Making evaluations based on measurements |  |  |
|  | Making improvements via the rapid-cycle method |  |  |
|  | Establishing whether changes lead to actual improvement |  |  |
|  | Having constructive meetings |  |  |
|  | Engaging in constructive discussions |  |  |
|  | Jointly reaching decisions |  |  |
|  | Turning decisions into actions to change |  |  |
|  | Applying the Process Redesign principles when redesigning a process |  |  |
|  | Reducing the turnaround time as much as possible in our hospital |  |  |
|  | Reducing the total hospitalization time as much as possible in our hospital |  |  |
|  | Translation of best practices of others |  |  |
|  | Converting (evidence-based) guidelines into standardized daily processes |  |  |
|  | Transferring knowledge and skills to colleagues involved in the process |  |  |
|  | Actively involving key persons and main stakeholders in the processes of change |  |  |

| 18 | **How do you assess the increase of skills in your team by your participation in the quality collaborative?**  **The skill to…** |  | 1 t/m 5 |
| --- | --- | --- | --- |
|  | Analyzing the current processes |  |  |
|  | Mapping out processes |  |  |
|  | Drawing connections between the different steps in the process |  |  |
|  | Identifying bottlenecks/problems |  |  |
|  | Identifying the causes of bottlenecks/problems |  |  |
|  | Specifying shared objectives for the whole chain |  |  |
|  | Generating solutions to bottlenecks/problems |  |  |
|  | Performing measurements |  |  |
|  | Making evaluations based on measurements |  |  |
|  | Making improvements via the rapid-cycle method |  |  |
|  | Establishing whether changes lead to actual improvement |  |  |
|  | Having constructive meetings |  |  |
|  | Engaging in constructive discussions |  |  |
|  | Jointly reaching decisions |  |  |
|  | Turning decisions into actions to change |  |  |
|  | Applying the Process Redesign principles when redesigning a process |  |  |
|  | Reducing the turnaround time as much as possible in our hospital |  |  |
|  | Reducing the total hospitalization time as much as possible in our hospital |  |  |
|  | Translation of best practices of others |  |  |
|  | Converting (evidence-based) guidelines into standardized daily processes |  |  |
|  | Transferring knowledge and skills to colleagues involved in the process |  |  |
|  | Actively involving key persons and main stakeholders in the processes of change |  |  |
|  | Other skills that have improved but are not mentioned? |  |  |

| 19 | **Which activities contributed the most to increase of these skills in you and/or your team?** |
| --- | --- |

1. This part of the questionnaire is supplementary material to the paper Weggelaar-Jansen, A.M., Wijngaarden, J., & Slaghuis, S. S. (2015). Do quality improvement collaboratives’ educational components match the dominant learning style preferences of the participants?. *BMC Health Services Research*, *15*(1), 239. [↑](#footnote-ref-2)
